# Dental Department, University Iowa

**Published:** 1888-11

**Authors:** 


### DENTAL DEPARTMENT OF THE UNIVERSITY OF IOWA.

We call attention to the announcement of the Dental Department University of Iowa, located at Iowa City. The department has been thoroughly organized by the new Dean, Dr. Hunt, and the fall term opens out with bright prospects. A full corps of teachers have been elected, and the seventh annual session promises to be the best ever held. Sixty students have matriculated up to date, Oct. 12, and ten more have been heard from that will come in later. Fifty-six was the largest class previous to this one.

